# Neonicotinoid insecticides limit the potential of spiders to re-colonize disturbed agroecosystems when using silk-mediated dispersal

**DOI:** 10.1038/s41598-019-48729-6

**Published:** 2019-08-22

**Authors:** Milan Řezáč, Veronika Řezáčová, Petr Heneberg

**Affiliations:** 1 Biodiversity Lab, Crop Research Institute, Drnovská 507, Prague, CZ-16106 Czechia; 2 Czech Academy of Sciences, Institute of Microbiology, Vídeňská 1083, Prague, CZ-142 20 Czechia; 3 Charles University, Third Faculty of Medicine, Ruská 87, Prague, CZ-100 00 Czechia

**Keywords:** Animal behaviour, Entomology, Natural hazards

## Abstract

Agroecosystems are subject to regular disturbances that cause extinction or migration of much of their fauna, followed by recolonization from surrounding refuges. In small-sized aeronaut spiders, such recolonization is potentiated by their ability to rappel and balloon. These are complex behaviors that we hypothesized to be affected by neurotoxins, namely, neonicotinoids. We tested this hypothesis using two common farmland spider species, *Oedothorax apicatus* (Linyphiidae) and *Phylloneta impressa* (Theridiidae). The spiders were topically exposed by dorsal wet application or tarsal dry exposure to commercial neonicotinoid formulations Actara 25 WG, Biscaya 240 OD, Mospilan 20 SP and Confidor 200 OD at concentrations that are recommended for application in agriculture. Contact exposure to neonicotinoids suppressed the ability of spiders to produce the major ampullate fiber and anchor it to the substratum by piriform fibrils. Contact exposure to neonicotinoids also suppressed the ballooning behavior that was manifested by climbing to elevated places, adopting a tiptoe position and producing silk gossamer in the wind. Impaired ability of affected common farmland spiders to quickly recolonize disturbed agroecosystems by silk-mediated dispersal may explain their decline in multiple farmland ecosystems, in which neonicotinoids are applied.

## Introduction

Agroecosystems are characterized by regular disturbances that cause extinction or migration of much of their fauna^1–4^. Therefore, these ecosystems must be repeatedly recolonized from surrounding refuges^5–8^. Spiders are among the most abundant predators in various agroecosystems and therefore serve as biological control agents in the entire range of agroecosystems, including orchards^9,10^, cabbage fields^11^, rice fields^12^, and wheat fields^13–15^. Migration abilities in combination with tolerance to agrochemicals are extremely important for the sustainable presence of these predators in dynamic agroecosystems. Therefore, agroecosystems are characterized by a relatively small number of highly dispersive species, which thrive despite (or thanks to) the disturbances but need to retain their dispersive abilities^16^. Sustainable presence of these spider species in agroecosystems is tightly related to their ability to re-colonize the habitats following the regular disturbances. Therefore, any agrochemicals that have adverse effects on the re-colonization abilities of spiders, such as those that suppress ballooning or rappelling, may have detrimental effects on the presence of the few species that managed to adjust to these hostile environments.

Schmidt *et al*.^17^ found that although the spider density in conventional winter wheat *Triticum aestivum* L., 1753 fields was positively related to the percentage of non-crop habitats; this relationship was absent when considering organic winter wheat fields. Their observation directed our attention to the fact that ballooning, as a characteristic complex behavior, might be affected by neurotoxic agrochemicals. Although information on adverse effects of the exposure to biocides on the ability to rappel and balloon is lacking, the exposure to biocides alters web size and/or web design^18–23^. The most widely used insecticides are currently neonicotinoids^24^. These neuroactive insecticide compounds compete with acetylcholine in binding to acetylcholine receptors of spiders^25,26^, but the sensitivity of spider acetylcholine receptors to neonicotinoids is lower than that of their insect orthologs^27^. In contrast to acetylcholine, acetylcholinesterase cannot break down neonicotinoids. The cells exposed to neonicotinoids are overstimulated, which may cause a wide array of neurological effects and eventually paralysis and death^28^.

Spiders cannot fly actively but small-sized aeronaut spiders use their silk as a balloon for passive transport by the wind. The propensity for wind dispersal of individual spider species is directly linked to their ability to colonize uniform agricultural landscapes^29^. The effectiveness of the dispersal by wind-mediated passive transport is inversely linked to fragmentation of semi-natural habitats^30^, increasing cover of arable land^31^, decreasing share of semi-natural habitats^32^, and increasing size of arable field blocks^16^. For example, the number of immigrating wolf spiders *Rabidosa rabida* (Walckenaer, 1837) to local populations recently decreased more than seven-fold, likely because of habitat fragmentation and ineffective undirected aerial dispersal in fragmented landscapes^30^. However, we note that some species, such as *Tenuiphantes tenuis* Blackwall, 1852, *Oedothorax apicatus* (Blackwall, 1850), and *Pachygnatha clercki* Sundevall, 1823, thrive in landscapes dominated by arable fields, with abundances even increasing with the proportion of arable fields^33^. Two basic types of wind-mediated dispersal are distinguished in spiders: 1) Ballooning, in which a spider climbs to some elevated spot, stretches the legs, raises the abdomen and releases major ampullate and piriform silk threads from the spinnerets to be carried by the wind to another spot; and 2) rappelling, in which a spider bridges from one elevated spot to another employing the wind and dropping a dragline (major ampullate fiber) attached to the first spot by an attachment disc (piriform fibers). Ballooning spiders can migrate for several km, whereas rappelling allows migration of only a few meters^34^. In agricultural landscapes, the ballooning behavior of spiders that are linked to arable field margins has increased risks as the fields serve as reproductive sinks; individuals of such species are unable to reproduce when landing at these ubiquitous unfavorable destinations^35,36^.

In the present study, we hypothesized the following: 1) contact exposure to neonicotinoids suppresses the ability of spiders to produce the major ampullate fiber and anchor it to the substratum by piriform fibrils; and 2) contact exposure to neonicotinoids suppresses the ballooning behavior that is manifested as climbing to elevated places, adopting a tiptoe position and producing silk gossamer in the wind. Combined, such effects would limit the affected small-sized aeronaut spiders from quickly recolonizing disturbed agroecosystems after their regular disturbances.

## Materials and Methods

### Model organisms

As model species, we used the first nymphal instars of the cobweb spider *Phylloneta impressa* (L. Koch, 1881) (Araneae: Theridiidae) that were collected from maternal nests in late spring and adult females of the money spider *Oedothorax apicatus* (Blackwall, 1850) (Araneae: Linyphiidae) that were collected in early spring. We used a single nymphal instar to ensure reproducibility and uniformity of observed responses. These species represent abundant predators of central European agroecosystems and readily perform the ballooning behavior and silk fiber production. The distribution of *O. apicatus* is centered to arable fields^9^, whereas *P. impressa* is characteristic for orchards, arable fields and grasslands^10^. We collected the study individuals individually a few days before the experiments. We acclimated the study individuals for at least a week in controlled conditions at 22 °C and 80% humidity with a natural light/dark regimen and fed them ad libitum non-flying mutants of *Drosophila melanogaster* Meigen, 1830 twice a week.

### Tested neonicotinoids

**Figure 1:**
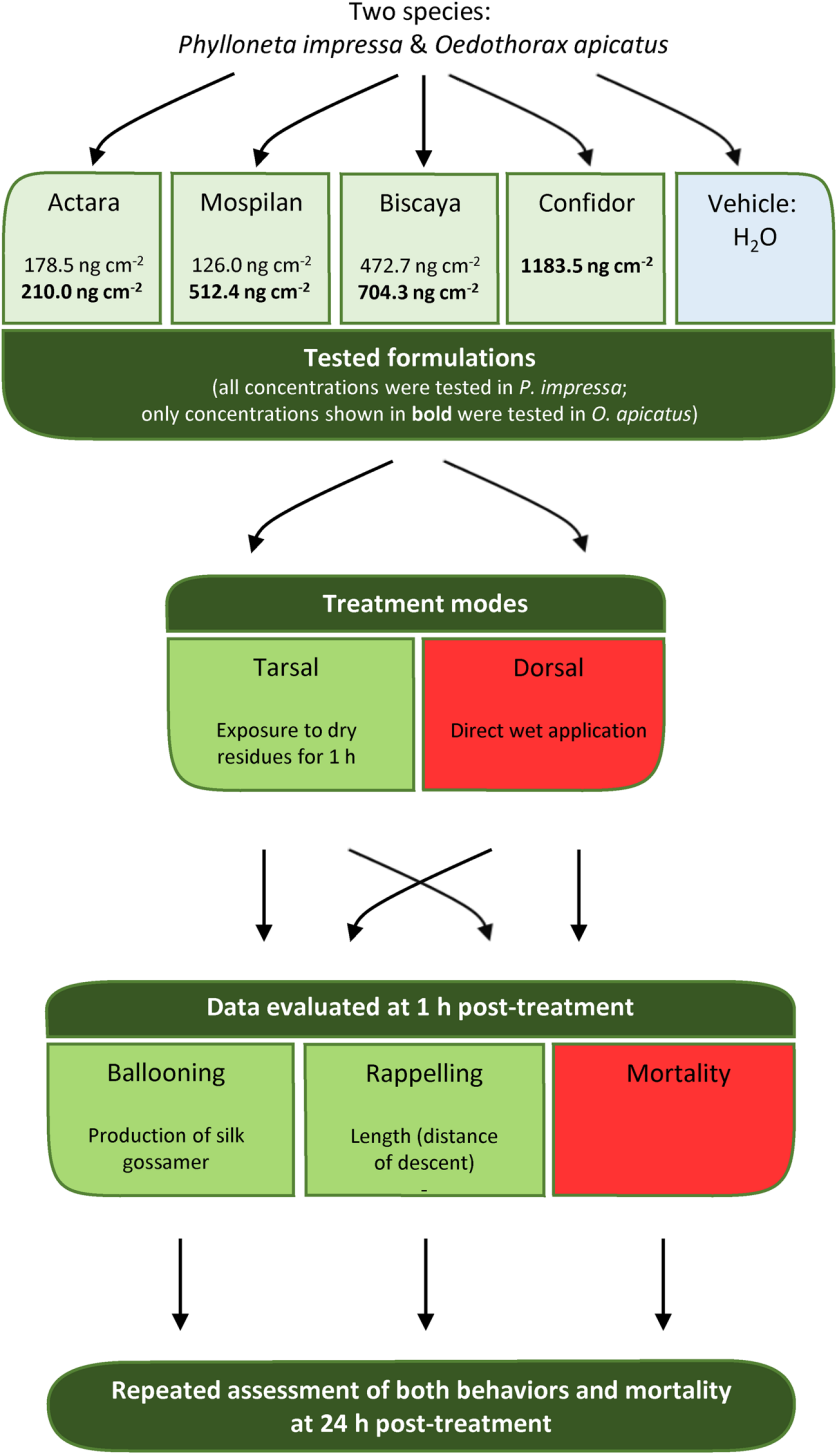
Scheme of the experimental setup.

We tested the effects of four neonicotinoids (imidacloprid, thiamethoxam, acetamiprid and thiacloprid) in formulations that are commonly used to spray crops to eliminate pest insects. Imidacloprid was formulated as Confidor 200 OD (Bayer CropScience, Monheim, Germany), which contained 19.3% of the active substance with suggested application of 600 ml ha^−1^. Thiacloprid was formulated as Biscaya 240 OD (Bayer CropScience, Monheim, Germany), which contained 22.97% of the active substance with suggested application of 200–300 ml ha^−1^. Thiamethoxam was formulated as Actara 25 WG (Syngenta Crop Protection, Basel, Switzerland), which contained 25% of the active substance with suggested application of 70–80 ml ha^−1^. Acetamiprid was formulated as Mospilan 20 SP (Nippon Soda Co., Tokyo, Japan), which contained 20% of the active substance with suggested application of 60–250 ml ha^−1^. As a vehicle and mock control, we used distilled water. Besides the active compound content, the exact composition of commercial formulations of the tested neonicotinoids was undisclosed. However, the farmers do not use pure chemicals but these commercial mixtures. Therefore, the use of distilled water as a control could be considered a limitation of the present study but clearly represents the effects of sham treatment. Each treatment group contained at least ten individuals. Reflecting two major ways of contact with biocides under field conditions, we either sprayed the commercial formulations of neonicotinoids (or distilled water) directly to the dorsal side of the body or allowed the spiders to walk over contaminated surfaces for tarsal contact with dry residues. To apply the commercial formulations of neonicotinoids, we diluted them to the maximum and minimum recommended concentrations for their use under field conditions (Confidor had a single recommended concentration) and sprayed them at 4.2 μl cm^−2^ using a Potter Precision Laboratory Spray Tower (Burkard Scientific, Uxbridge, UK). The concentrations used were 178.5 ng cm^−2^ and 210.0 ng cm^−2^ for Actara 25 WG, 472.7 ng cm^−2^ and 704.3 ng cm^−2^ for Biscaya 240 OD, 1183.5 ng cm^−2^ for Confidor 200 OD, and 126.0 ng cm^−2^ and 512.4 ng cm^−2^ for Mospilan 20 SP. To treat *P. impressa*, we used both the highest and lowest recommended concentrations (further termed high and low); to treat *O. apicatus*, we used the high concentrations only. For the dorsal application, we sprayed the commercial formulations of neonicotinoids directly on the dorsal surface of the spiders and moved them immediately to clean 24-well plates. For the tarsal exposure to dry residues, we sprayed empty wells with commercial formulations of neonicotinoids at concentrations identical to those used for the dorsal application, and we introduced the spiders only later, after the microdrops on the bottom of dishes dried. We recorded the acute mortality after a one-hour exposure and tested the sublethal effects only on individuals that survived the first post-application hour. To check the ability to recover from the effect of commercial formulations of neonicotinoids, we repeated the same assessment with the identical animals 24 hours after the application of commercial formulation of neonicotinoids. Scheme of the experimental setup is provided in Fig. 1.

### Ballooning behavior

One hour after acclimation and treatment with neonicotinoids, we stimulated ballooning by simulating a slight breeze at 2 m s^−1^ from a ventilator and by placing tested spiders in dishes with vertical wooden sticks as described by Pétillon *et al*.^37^. This environment stimulated the control spiders to climb to the very tops of sticks, where they adopted the tiptoe position and produced silk gossamer from their abdomens. We recorded the ratio of ballooning animals *versus* those that stayed on the bottom of the dish. The ballooning stimulation lasted 15 min or until the ballooning was manifested. Any ballooning displayed during the 15-min evaluation period was counted as a positive record.

### Rappelling behavior

One hour after acclimation and treatment with neonicotinoids, we stimulated rappelling by placing the spiders on 10 × 10 cm glass square plates that were positioned atop one meter long sticks. Control spiders regularly attached their dragline (major ampullate fiber) to the glass plate by attachment discs, i.e., sticky piriform fibers^38^. Failure to display rapelling manifests by problems with anchoring the dragline to the glass plate and/or with the production of the dragline itself. We recorded the length (distance) of descents down to the bottom while being secured by dragline, which also included the requirement of a successful anchor of the dragline to the substratum. The rapelling stimulation lasted 15 min or until the rapelling was manifested. Due to the experimental design, we only recorded the length of the dragline resulting from the first rapelling attempt. The spider individuals that were used in the rappelling experiment were not identical with those used in the ballooning experiment.

### Statistical analyses

Each spider was treated individually, considered a single experimental unit. Data are shown as the mean ± SE unless stated otherwise. The rappelling data with normal distribution and equal variance were tested by one-way ANOVA; other data were tested by Kruskal-Wallis one-way ANOVA on ranks. The comparisons of 1 h vs 24 h post-treatment experiments with outcomes of normally distributed data were performed by *t*-test; Mann-Whitney Rank Sum Test was used to test other data. The data on ballooning and mortality were tested by χ^2^ test with Yates correction for continuity, followed by a post-hoc power analysis. Because previously obtained data on the effects of neonicotinoids on rappelling and ballooning of spiders were not available, we chose the size of the tested cohorts ad hoc and performed a post-test power analysis on the obtained data. The analyses were conducted in SigmaPlot 12.0.

## Results

### Mortality

**Figure 2:**
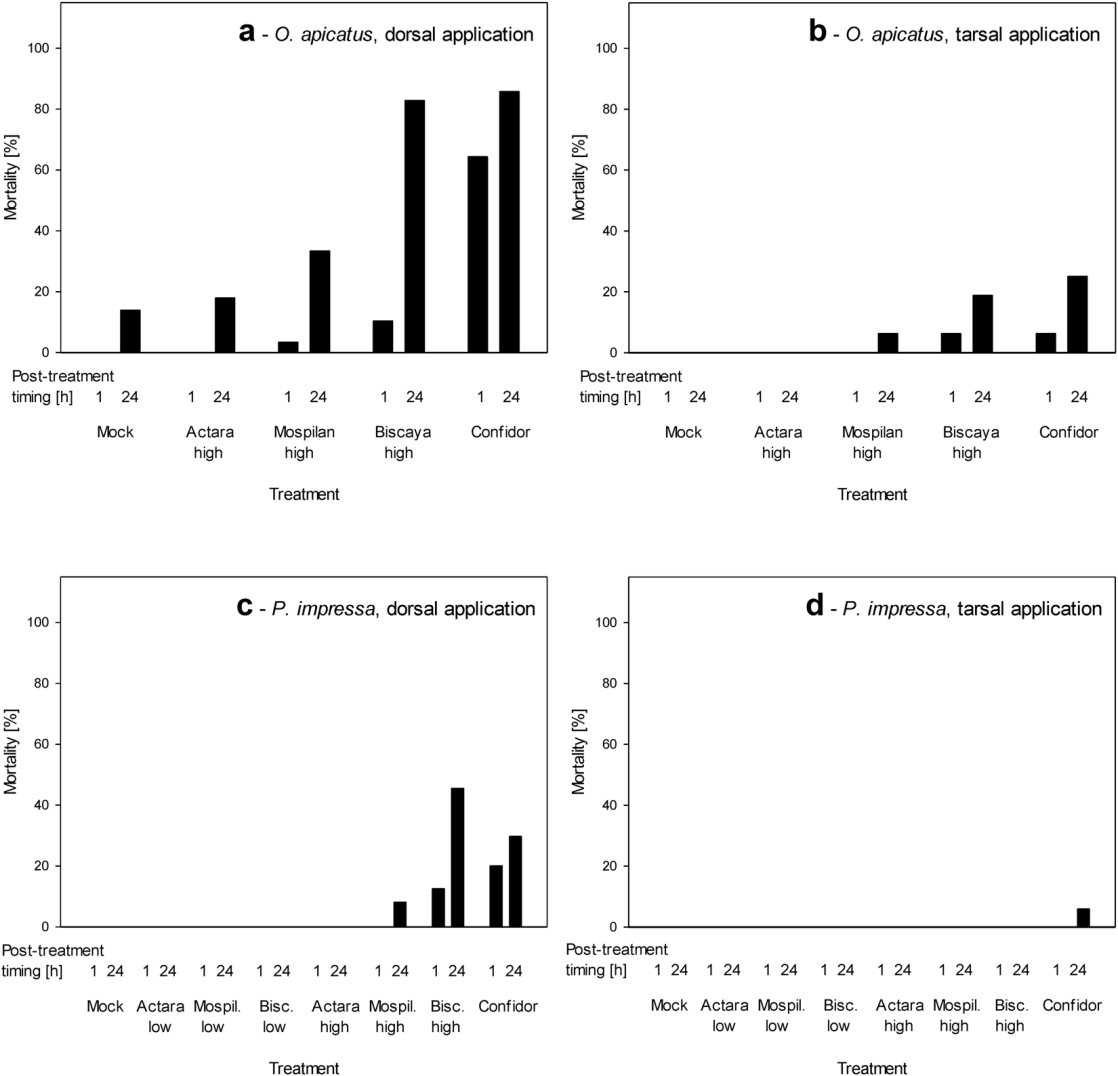
Effects of treatments with neonicotinoid insecticides on mortality of *Oedothorax apicatus* (**a,b**) and *Phylloneta impressa* (**c,d**) during 1 h and 24 h post-exposure periods. The data are shown as the proportion of spiders that died during the 1 h or 24 h post-exposure interval. The neonicotinoids were either applied wet dorsally (**a,c**) or the spiders were exposed to their dry residues tarsally (**b,d**).

The neonicotinoids differed in their effects with regards to the site of contact exposure (dorsal wet application vs. tarsal exposure to dry residues; Table 1) and with regards to the time elapsed from the treatment (1 h vs 24 h; Table S1). Dorsal treatment with all four tested neonicotinoids significantly increased mortality of both tested spider species when evaluated at either 1 h or 24 h after the treatment (Fig. 2). The mortality increased significantly when evaluated after the 24 h interval for *O. apicatus* treated with Mospilan or Biscaya (Fig. 2a, Table S1). For both spider species, the strongest effects were obtained with dorsally applied Confidor, followed by those of Biscaya, Mospilan and Actara; only Actara did not induce mortality in one of the tested species, *P. impressa* (Fig. 2c). For Biscaya, Mospilan and Actara, we tested the concentrations that were at the upper and lower limits of their recommended dilutions for use in agriculture. We tested the high concentrations on both spider species, whereas we tested the low concentrations on *P. impressa* only. In contrast to the effects of the high concentrations, dorsal application of the lower concentrations did not induce any mortality of *P. impressa* (Fig. 2c). When the neonicotinoids were applied using the mode of tarsal exposure to dry residues, the trends in effects were similar. However, the effects were much weaker than those induced by dorsal application, with only up to 25% mortality in *O. apicatus* (Fig. 2b) and negligible mortality of *P. impressa* following any treatment by mode of tarsal exposure to dry residues (Fig. 2d).

**Table 1:** Effects of treatments with neonicotinoid insecticides on the mortality of *Oedothorax apicatus* and *Phylloneta impressa* during 1 h and 24 h post-exposure periods.

Species	Application	χ^2^	*D*_*f*_	*P*	Power (at α = 0.05) *P*
1 h post-treatment					
*O. apicatus*	Dorsal	67.199	4	<0.001	1.000
*O. apicatus*	Tarsal	6.280	4	>0.05	0.480
*P. impressa*	Dorsal	31.281	7	<0.001	0.996
*P. impressa*	Tarsal	N/T	7	N/T	N/T (all alive)
24 h post-treatment					
*O. apicatus*	Dorsal	59.514	4	<0.001	1.000
*O. apicatus*	Tarsal	18.333	4	=0.001	0.955
*P. impressa*	Dorsal	55.312	7	<0.001	1.000
*P. impressa*	Tarsal	8.818	7	>0.05	0.541

Only the individuals that survived the 1 h or 24 h post-exposure period were included in further experiments that focused on ballooning and rappelling. The data were tested by χ^2^ test, followed by post-hoc power analysis.

### Ballooning

**Figure 3:**
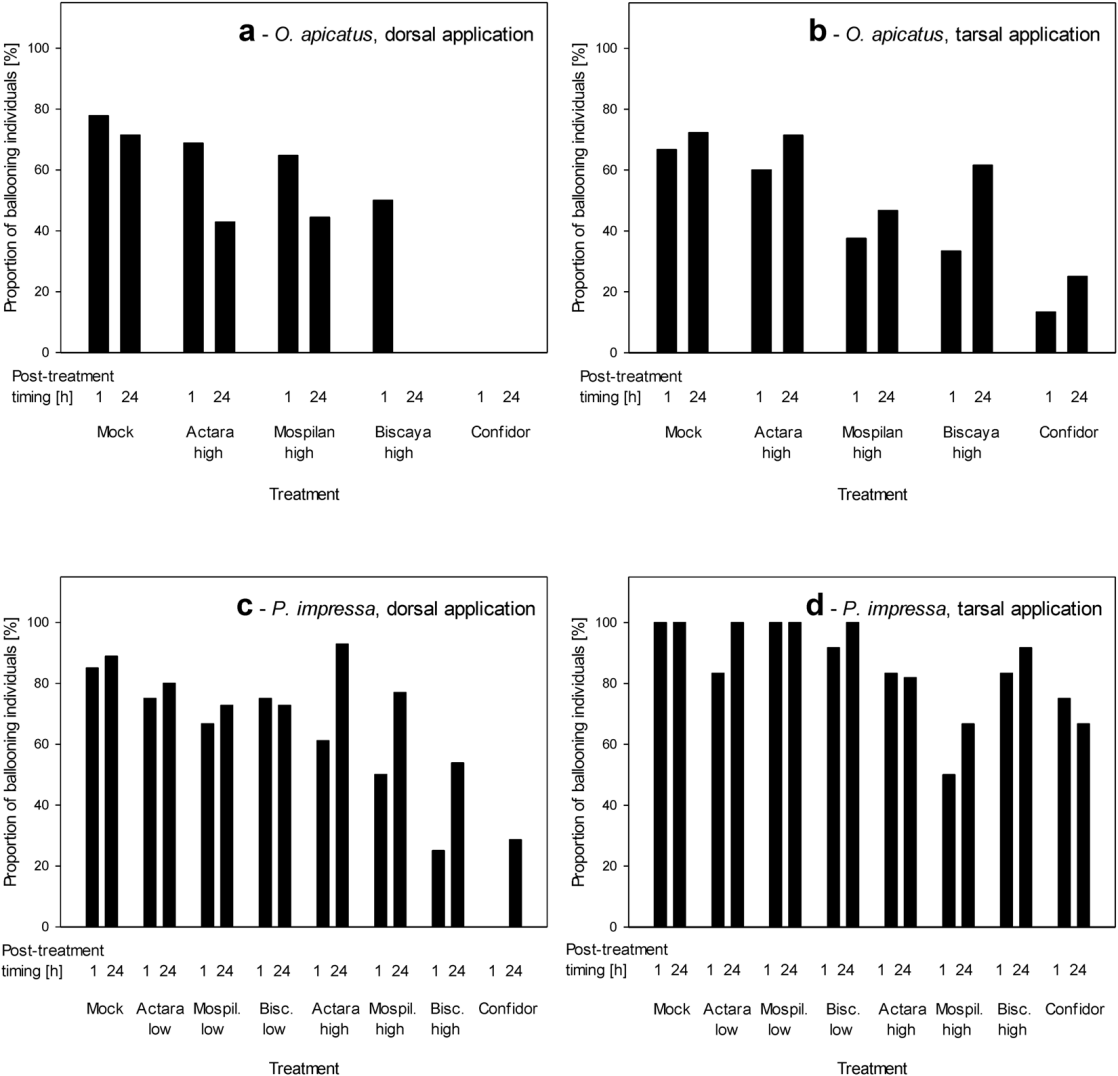
Effects of treatments with neonicotinoid insecticides on the ballooning activity of *Oedothorax apicatus* (**a,b**) and *Phylloneta impressa* (**c,d**) after 1 h and 24 h post-exposure periods. The data are shown as the proportion of spiders that ballooned when tested after 1 h or 24 h following exposure to neonicotinoids. The neonicotinoids were either applied wet dorsally (**a,c**) or the spiders were exposed to their dry residues tarsally (**b,d**).

The effects neonicotinoids on ballooning behavior differed dramatically with respect to the site of contact exposure (dorsal wet application vs. tarsal exposure to dry residues; Table 2), whereas the time elapsed from the treatment (1 h vs 24 h) had no major effect (Table S2). Dorsal treatment with all four neonicotinoids significantly decreased the ballooning activity of *P. impressa* when evaluated at either 1 h or 24 h after the treatment (Fig. 3). The ballooning activity decreased the most following the treatment with Biscaya, followed by decreases with Mospilan, Confidor and Actara, all at the highest concentrations tested (Fig. 3a,c). At the concentrations at the lower limits of their recommended dilutions for use in agriculture, Biscaya, Mospilan and Actara had only marginal effects on the ballooning activity of *P. impressa* when applied dorsally (Fig. 3c). When the neonicotinoids were applied using the mode of tarsal exposure to dry residues, the trends in effects were similar, but the effects were in some cases weaker than those induced by dorsal application. For example, the 1 h exposure to the high Biscaya concentrations resulted in ballooning of 33% of *P. impressa* individuals when applied dorsally but 83% of *P. impressa* individuals when exposed to dry residues tarsally (Fig. 3b,d). Tarsal exposure to dry residues of Biscaya, Mospilan and Actara at the lower limit of their recommended dilutions for use in agriculture had only marginal effects on the ballooning activity of *P. impressa* (Fig. 3d).

**Table 2:** Effects of treatments with neonicotinoid insecticides on the ballooning activity of *Oedothorax apicatus* and *Phylloneta impressa*.

Species	Application	χ^2^	*D*_*f*_	*P*	Power (at α = 0.05) *P*
1 h post-treatment					
*O. apicatus*	Dorsal	2.988	3	>0.05	0.264
*O. apicatus*	Tarsal	12.035	4	=0.017	0.807
*P. impressa*	Dorsal	36.329	7	<0.001	0.999
*P. impressa*	Tarsal	15.270	7	=0.033	0.829
24 h post-treatment					
*O. apicatus*	Dorsal	2.540	3	>0.05	0.228
*O. apicatus*	Tarsal	8.664	4	>0.05	0.639
*P. impressa*	Dorsal	20.855	7	=0.004	0.944
*P. impressa*	Tarsal	16.031	7	=0.025	0.852

The data were tested by χ^2^ test, followed by post-hoc power analysis.

### Rappelling

**Figure 4:**
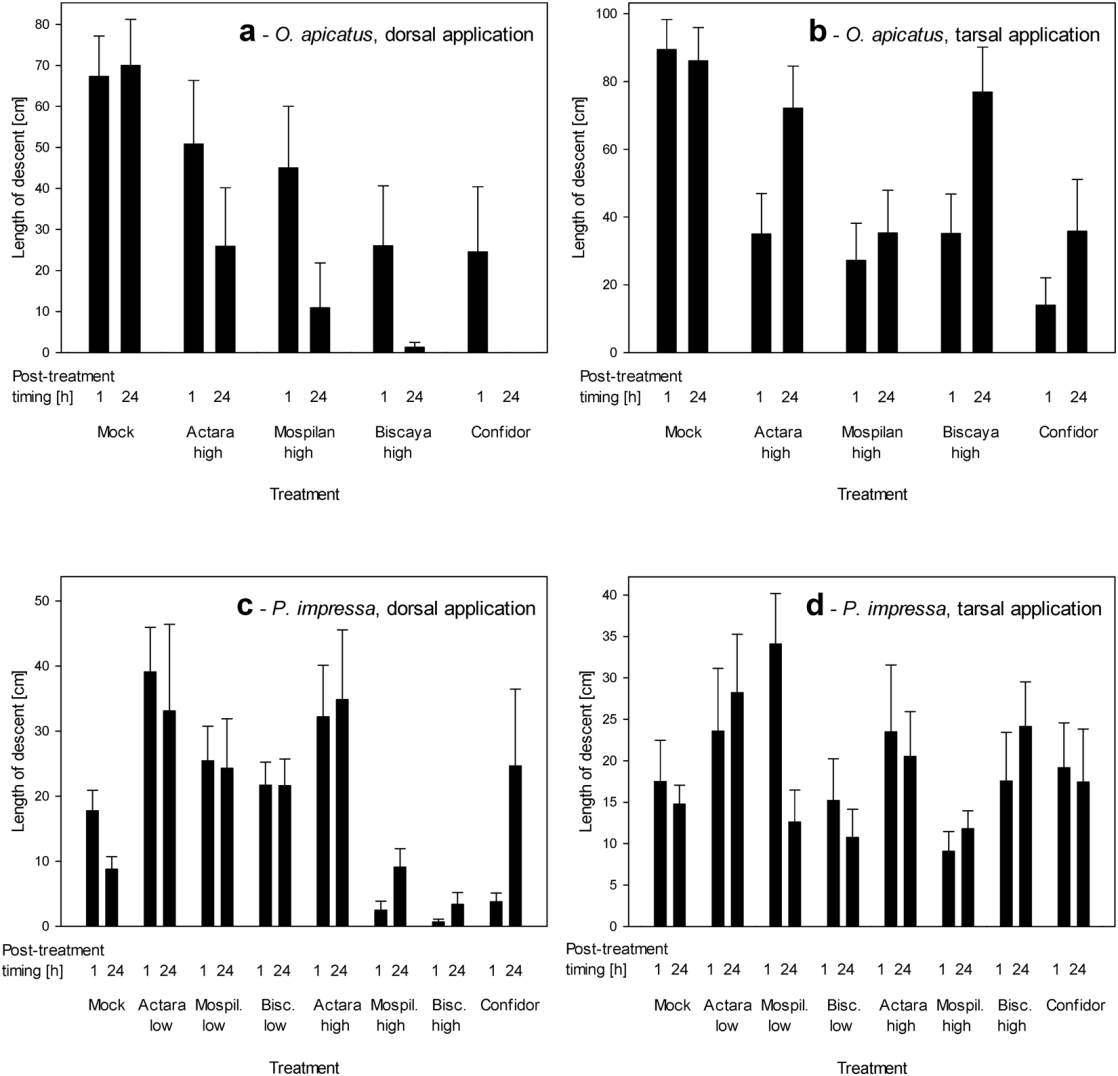
Effects of treatments with neonicotinoid insecticides on the rappelling activity of *Oedothorax apicatus* (**a,b**) and *Phylloneta impressa* (**c,d**) after 1 h and 24 h post-exposure periods. The data are shown as the length of descent that the spiders performed while being secured by a dragline when tested after 1 h or 24 h following exposure to neonicotinoids. The neonicotinoids were either applied wet dorsally (**a,c**) or the spiders were exposed to dry residues tarsally (**b,d**).

Rappelling behavior showed high variability within the tested groups of spiders. We observed significant effects of the administration of neonicotinoids only at 1 h post-exposure time in tarsally exposed *O. apicatus* and dorsally treated *P. impressa* (Table 3). Some variability was associated with the time elapsed from the treatment (1 h vs 24 h), particularly concerning the Mospilan treatments, which, in some cases, displayed stronger effects following the longer time interval than those after the shorter interval (Fig. 4, Table S3). When considering dorsal treatment, we noticed a decreased length of descent following the exposure to high concentrations of Mospilan, Biscaya and Confidor in both spider species (Fig. 4a,c). However, in *P. impressa*, low concentrations of Actara, Biscaya or Mospilan did not induce significant effects (Fig. 4c), and rappelling distance increased for *P. impressa* after 24 h of exposure to high concentration Confidor (Fig. 4c). The length of descent decreased following tarsal exposure to dry residues of all of the neonicotinoids, but only when *O. apicatus* was treated (Fig. 4b, Table 3); tarsal exposure of *P. impressa* did not induce any effects concerning the rappelling behavior (Fig. 4d, Table 3).

**Table 3:** Effects of treatments with neonicotinoid insecticides on the rapelling activity of *Oedothorax apicatus* and *Phylloneta impressa*.

Species	Application	Shapiro-Wilk normality test *P*	Levene’s equal variance test *P*	*F* / *H*	*D*_*f*_	*P*	Dunn’s post-test vs. mock *P*; treatment
1 h post-treatment							
*O. apicatus*	Dorsal	<0.05	N/T	9.145	4	0.05	
*O. apicatus*	Tarsal	<0.05	N/T	21.006	4	<0.001	<0.05 Actara, high; <0.05 Mospilan, high; <0.05 Biscaya, high; <0.05 Confidor
*P. impressa*	Dorsal	<0.05	N/T	65.804	7	<0.001	N/S Actara, high; <0.05 Mospilan, high; <0.05 Biscaya, high; <0.05 Confidor; N/S Actara, low; N/S Mospilan, low; N/S Biscaya, low
*P. impressa*	Tarsal	<0.05	N/T	13.680	7	>0.05	
24 h post-treatment							
*O. apicatus*	Dorsal	<0.05	N/T	19.031	4	<0.001	N/S Actara, high; <0.05 Mospilan, high; N/S Biscaya, high; <0.05 Confidor
*O. apicatus*	Tarsal	<0.05	N/T	11.311	4	0.023	N/S Actara, high; N/S Mospilan, high; N/S Biscaya, high; N/S Confidor
*P. impressa*	Dorsal	<0.05	N/T	27.891	7	<0.001	N/S Actara, high; N/S Mospilan, high; N/S Biscaya, high; N/S Confidor; N/S Actara, low; N/S Mospilan, low; N/S Biscaya, low
*P. impressa*	arsal	<0.05	N/T	10.769	7	>0.05	

The data with normal distribution and equal variance were tested by one-way ANOVA; other data were tested by Kruskal-Wallis one-way ANOVA on ranks.

## Discussion

Ballooning and rappelling are complex activities that require coordination of the secretion of numerous glands together with the ability of a spider to assess properly the conditions that support the success of such behavior. For ballooning, spiders produce two types of silk fibers, long, thick ones produced from the major ampullate spinning glands and thin, sticky ones produced from the piriform spinning glands. For rappelling, spiders must produce an “anchor-rope” system that allows the safe descent from elevated spots^38^. Silk secretion and spinning are easily disrupted by biocides as shown by numerous authors when evaluating the size and/or design of webs produced by orb weavers^18–23,39^. In orb-weavers, the effects of neonicotinoids include the down-regulation of silk strength, toughness, and extensibility, the alteration of mechanical properties, structure, and amino acid composition, and the down-regulation of the silk protein MaSp2^39^. However, the present study is the first to address the effects of biocides on ballooning and rappelling, which are key tools that are responsible for the ability of spiders to re-colonize disturbed agroecosystems.

In the present study, we provide the first evidence that some of the neonicotinoid formulations that are used in agriculture have adverse effects on the ballooning and rappelling of spiders. We showed that there exist multiple conditions, under which the contact application of any of the four tested compounds compromised the dispersal abilities of both examined spider species. The tested compounds often displayed acute mortality. However, the mortality was not consistently associated with all tested combinations. There was no mortality following tarsal exposure of *P. impressa*, the most of dorsal treatments of *P. impressa* and even a few treatments of *O. apicatus*. All tested neonicotinoids decreased the number of spiders that displayed ballooning behavior. Additionally, all tested compounds inhibited in some of their formulations rappelling activity. In some experimental settings, particularly the lower concentrations often displayed the opposite effect on rappelling. In *P. impressa*, even high Actara concentrations increased rappelling distance. It remains to be tested, whether any such signs of increased dispersal may represent adaptive responses to the insecticides. The effects were observed nearly instantly, and the extent of these effects did not change much between 1 h and 24 h following topical application of neonicotinoids, with a couple of exceptions.

The present study leads to several important and unexpected conclusions. All four tested neonicotinoids caused adverse effects on ballooning and rappelling when applied at concentrations that are recommended by the manufacturers for use in agriculture. This is true not only for Confidor, which is currently considered the most problematic among the four tested neonicotinoids with previously confirmed adverse effects on spiders^40,41^. We found severe sublethal effects associated also with the other three neonicotinoids. Among the four tested neonicotinoids, based on previous studies, Actara is suggested as less toxic to predatory arthropods^42–44^, including spiders^45–47^. Similar results were observed for Mospilan with regards to spiders^48^ and because of the supposedly negligible toxicity, Mospilan is even subject to less strict regulation in the European Union. However, multiple independent experiments that were performed in the present study did not support the claims on negligible toxicity of Mospilan.

In contrast to effects of the highest recommended doses of neonicotinoids, the lowest effective concentrations, which were recommended by the manufacturers, were associated with substantially milder (and sometimes even absent or slightly positive) effects on the ballooning and rappelling behaviors. Based on this result, the previously suggested window for neonicotinoids use that would continue to allow the benefits of insecticidal effects but avoid unnecessary damage to off-target arthropods e.g.^49,50^, such as spiders, might indeed exist. However, this window is apparently much smaller than previously thought and much smaller than proposed by disclosed outcomes of toxicity tests, which are notoriously limited by the inclusion of only a few easy-to-maintain arthropod species. Moreover, the few existing ecotoxicity tests that focus on spiders include tests of survival, food consumption and vaguely defined behavioral changes^51–55^. In addition, although the observed effects were acute and manifested within the first post-treatment hour, these effects lasted for at least 24 h following the contact treatment. In combination with increased mortality during the 24 h post-treatment period (in comparison with the 1 h post-treatment period), the observed effects likely persisted for a time period sufficient to affect the dispersal of affected spiders, which is highly important given that the study species represented common farmland spiders^9,10^. Thus, if neonicotinoids have adverse effects on these species when applied at concentrations recommended by the manufacturers, the widespread application of neonicotinoids by farmers affects dispersal behaviors and mortality of these important farmland predators. Because high dispersal ability of the examined common farmland spider species is a prerequisite for their distribution across a wide range of disturbed agrocenoses, harming their dispersal ability indicates that we may adversely affect their long-term population trends and ability to re-colonize the periodically disturbed sites.

When comparing the effects of dorsal wet application and tarsal exposure to dry residues of neonicotinoids, we have shown that the spiders were affected the most by the direct application of wet residues. In contrast, later exposure to dry residues had much weaker effects. These differences manifested prominently when the mortality was tracked (Fig. 2) but much less when the sublethal effects were tracked (Figs 3–4). However, it is important to note that the real field exposure consists not only of acute exposure to the neonicotinoids during their application (mimicked by dorsal wet application) but includes also immediately starting chronic exposure to the dry residues (mimicked only to a short-term extent by the tarsal exposure). We assume that the effects of the two exposure modes are additive. While we observed the full extent of the effects of acute exposure, further studies need to address the effects of chronic exposure. Particularly wandering spiders are in a danger of a chronic contact with dry residues of neonicotinoids. In contrast to them, the orb-weavers may be affected rather by the acute exposure only, as they are not in permanent contact with soil or plant surfaces.

To conclude, we provided multiple points of evidence that contact exposure to neonicotinoids changed propensity of spiders for dispersal behaviors and induced variation in rappelling distance. We found that all four tested neonicotinoids, including those with previously claimed negligible effects on spiders, severely inhibited both ballooning and rappelling behaviors of spiders when applied in concentrations recommended by the manufacturers for use in agriculture. Impaired ability of affected common farmland spiders to quickly recolonize disturbed agroecosystems after their regular disturbances may explain their decline in multiple farmland ecosystems, in which neonicotinoids are applied.

## Supplementary information

Supplementary Tables

## Data Availability

All data generated or analysed during this study are included in this published article (and its Supplementary Information files).
