# Supplementary material for: Neonicotinoid insecticides limit the potential of spiders to re-colonize disturbed agroecosystems when using silk-mediated dispersal
Source: Sci Rep. 2019 Aug 22;9:12272. doi: 10.1038/s41598-019-48729-6 (PMC6706445; doi:10.1038/s41598-019-48729-6)
Supplement: Supplementary file 1 — Supplementary Tables [file 41598_2019_48729_MOESM1_ESM.pdf]

SUPPLEMENTARY MATERIALS TO:

Neonicotinoid insecticides limit the potential of spiders to re-colonize disturbed agroecosystems when using silk-mediated dispersal

Milan Řezáč[1], Veronika Řezáčová[2], Petr Heneberg[1,3,*]

Authors' affiliations:

[1] Biodiversity Lab, Crop Research Institute, Drnovská 507, CZ-16106 Prague 6 – Ruzyně, Czechia

[2] Czech Academy of Sciences, Institute of Microbiology, CZ-142 20 Prague, Czechia

[3] Charles University, Third Faculty of Medicine, Prague, Czechia

[*] *Address correspondence to:* Petr Heneberg, Charles University, Third Faculty of Medicine, Ruská 87, CZ-100 00 Prague, Czechia, Tel. ++420 – 775 311 177, Fax ++420 – 267 162 710, E-mail: petr.heneberg@lf3.cuni.cz

Supplementary Tables

Table S1. Comparison of the effects of treatments with neonicotinoid insecticides on the mortality of *Oedothorax apicatus* and *Phylloneta impressa* during 1 h and 24 h post-exposure periods. The data were tested by χ^2 test, followed by post-hoc power analysis.

Species	Application	χ^2	D_f	P	Power (at α= 0.05) P	Treatment	N_{1h}	N_{24h}
1 h vs 24 h treatment								
O. apicatus	Dorsal	2.859	1	N/S	0.377	Mock	31	36
		N/T	1	N/T	N/T (zero dead)	Actara, high	28	28
		7.124	1	= 0.008	0.772	Mospilan, high	30	30
		27.718	1	< 0.001	1.000	Biscaya, high	29	29
		2.381	1	N/S	0.321	Confidor	28	28
O. apicatus	Tarsal	N/T	1	N/T	N/T (zero dead)	Mock	36	36
		3.514	1	N/S	0.452	Actara, high	30	28
		0.516	1	N/S	0.103	Mospilan, high	32	32
		1.286	1	N/S	0.191	Biscaya, high	32	32
		2.963	1	N/S	0.085	Confidor	32	32
P. impressa	Dorsal	N/T	1	N/T	N/T (zero dead)	Mock	38	34
		N/T	1	N/T	N/T (zero dead)	Actara, high	33	27
		0.859	1	N/S	0.142	Mospilan, high	33	25
		5.756	1	0.016	0.672	Biscaya, high	32	22
		0.286	1	N/S	0.078	Confidor	30	27
		N/T	1	N/T	N/T (zero dead)	Actara, low	23	19
		N/T	1	N/T	N/T (zero dead)	Mospilan, low	23	22
		N/T	1	N/T	N/T (zero dead)	Biscaya, low	23	21
P. impressa	Tarsal	N/T	1	N/T	N/T (zero dead)	Mock	24	24
		N/T	1	N/T	N/T (zero dead)	Actara, high	23	21
		N/T	1	N/T	N/T (zero dead)	Mospilan, high	24	24
		N/T	1	N/T	N/T (zero dead)	Biscaya, high	24	22
		0.012	1	N/S	0.048	Confidor	21	17
		N/T	1	N/T	N/T (zero dead)	Actara, low	24	20
		N/T	1	N/T	N/T (zero dead)	Mospilan, low	20	17
		N/T	1	N/T	N/T (zero dead)	Biscaya, low	23	21

Table S2. Comparison of the effects of treatments with neonicotinoid insecticides on the ballooning activity of *Oedothorax apicatus* and *Phylloneta impressa* after 1 h and 24 h post-exposure periods. The data were tested by χ^2 test, followed by post-hoc power analysis.

Species	Application	χ^2	D_f	P	Power (at α= 0.05) P	Treatment	N_{1h}	N_{24h}
1 h vs 24 h treatment								
		0.035	1	N/S	0.051	Mock	18	7
		0.499	1	N/S	0.101	Actara, high	16	7
O. apicatus	Dorsal	0.334	1	N/S	0.083	Mospilan, high	17	9
		N/T	N/T	N/T	0.048	Biscaya, high	16	1
		N/T	N/T	N/T	N/T (lethal)	Confidor	0	0
		0.000	1	N/S	0.047	Mock	18	18
		0.066	1	N/S	0.054	Actara, high	15	14
O. apicatus	Tarsal	0.023	1	N/S	0.050	Mospilan, high	16	15
		1.238	1	N/S	0.185	Biscaya, high	15	13
		0.004	1	N/S	0.048	Confidor	15	12
		0.016	1	N/S	0.049	Mock	20	18
		2.709	1	N/S	0.359	Actara, high	18	14
		1.311	1	N/S	0.194	Mospilan, high	18	13
P. impressa	Dorsal	1.458	1	N/S	0.211	Biscaya, high	16	13
		2.859	1	N/S	0.377	Confidor	15	14
		0.054	1	N/S	0.053	Actara, low	12	10
		0.019	1	N/S	0.049	Mospilan, low	12	11
		0.123	1	N/S	0.061	Biscaya, low	12	11
		N/T	1	N/T	N/T (zero neg.)	Mock	12	12
		0.207	1	N/S	0.070	Actara, high	12	11
		0.171	1	N/S	0.066	Mospilan, high	12	12
P. impressa	Tarsal	0.000	1	N/S	0.047	Biscaya, high	12	12
		0.005	1	N/S	0.048	Confidor	12	9
		0.457	1	N/S	0.097	Actara, low	12	11
		N/T	1	N/T	N/T (zero neg.)	Mospilan, low	11	9
		0.002	1	N/S	0.047	Biscaya, low	12	11

Table S3. Comparison of effects of treatments with neonicotinoid insecticides on the rappelling activity of *Oedothorax apicatus* and *Phylloneta impressa* after 1 h and 24 h post-exposure periods. The comparisons of 1 h vs 24 h post-treatment experiments with outcomes of a normal distribution were performed by t-test; Mann-Whitney Rank Sum Test was used to test other data.

Species	Application	Shapiro-Wilk normality test *P*	Levene's equal variance test *P*	*t* / *U*	*P*	Treatment	N_{1h}	N_{24h}
1 h vs 24 h treatment								
O. apicatus	Dorsal	< 0.05	N/T	100.000	N/S	Mock	15	14
		< 0.05	N/T	47.500	N/S	Actara, high	12	11
		< 0.05	N/T	26.500	= 0.005	Mospilan, high	13	11
		< 0.05	N/T	17.500	N/S	Biscaya, high	10	4
		< 0.05	N/T	14.000	N/S	Confidor	10	4
O. apicatus	Tarsal	< 0.05	N/T	159.500	N/S	Mock	18	18
		< 0.05	N/T	59.500	N/S	Actara, high	14	14
		< 0.05	N/T	115.500	N/S	Mospilan, high	16	15
		< 0.05	N/T	52.500	= 0.034	Biscaya, high	15	14
		< 0.05	N/T	82.500	N/S	Confidor	15	12
P. impressa	Dorsal	< 0.05	N/T	74.000	= 0.048	Mock	18	16
		< 0.05	N/T	96.500	N/S	Actara, high	15	13
		< 0.05	N/T	31.500	= 0.012	Mospilan, high	15	12
		< 0.05	N/T	66.500	N/S	Biscaya, high	16	9
		< 0.05	N/T	52.000	N/S	Confidor	15	13
		< 0.05	N/T	32.000	N/S	Actara, low	11	9
		< 0.05	N/T	45.500	N/S	Mospilan, low	11	11
		N/S	N/S	0.0168	N/S	Biscaya, low	11	10
P. impressa	Tarsal	< 0.05	N/T	67.000	N/S	Mock	12	12
		< 0.05	N/T	49.000	N/S	Actara, high	10	10
		< 0.05	N/T	39.500	N/S	Mospilan, high	12	12
		< 0.05	N/T	22.000	N/S	Biscaya, high	12	10
		< 0.05	N/T	67.500	N/S	Confidor	9	8
		< 0.05	N/T	40.000	N/S	Actara, low	12	9
		N/S	N/S	14.000	= 0.004	Mospilan, low	9	8
		< 0.05	N/T	0.720	N/S	Biscaya, low	11	10
